# HLA‐DR⁺ Tumor Cells Show an Association with a Distinct Immune Microenvironment and CD8⁺ T‐Cell Exhaustion in HBV‐Associated Hepatocellular Carcinoma

**DOI:** 10.1002/advs.202502979

**Published:** 2025-06-04

**Authors:** Jun‐Qing Chang, Yan Guo, Wen‐Jing Yuan, Yu‐Man Chen, Bo‐Wei Liu, Wen‐Tao Li, Xiang‐Ming Ding, Xu‐Dong Fu, Yu‐Han Lou, Zhuo‐Ran Chen, Xiao‐Ying Luo, Song‐Ze Ding, Bing‐Yong Zhang, Xiu‐Ling Li, Yong‐Zhi Hong, Shun‐Dong Cang, Dong‐Xiao Li, Ling Lan

**Affiliations:** ^1^ Department of Gastroenterology and Hepatology Henan Provincial People's Hospital; Zhengzhou University People's Hospital; Xinxiang Medical University Zhengzhou 450003 China; ^2^ Department of Oncology Henan Provincial People's Hospital; Zhengzhou University People's Hospital Zhengzhou 450003 China; ^3^ Department of Gastroenterology and Hepatology People's Hospital of Henan University Zhengzhou 450003 China; ^4^ Department of Breast Surgery Henan Provincial People's Hospital; Zhengzhou University People's Hospital Zhengzhou 450003 China; ^5^ Department of Gastroenterology and Hepatology the First Affiliated Hospital of Henan University Kaifeng 475001 China; ^6^ Department of Gastroenterology and Hepatology Henan No.3 Provincial People's Hospital Zhengzhou 450006 China; ^7^ Department of General Surgery Susong County Hospital of Traditional Chinese Medicine Susong 246501 China

**Keywords:** hepatitis B virus, hepatocellular carcinoma, HLA‐DR⁺ tumor cells, immunosuppressive, tumor microenvironment

## Abstract

Hepatocellular carcinoma (HCC) is a leading cause of cancer‐related deaths worldwide, with hepatitis B virus (HBV) as a major driver. Despite the pivotal role of viral infections in shaping the tumor microenvironment (TME), the mechanistic differences among HBV‐, hepatitis C virus (HCV)‐, and non‐B non‐C (NBNC)‐associated HCC remain poorly understood. By integrating the largest publicly available single‐cell RNA sequencing (scRNA‐seq) dataset of HCC (160 samples from 124 patients) with multi‐scale protein‐level validation using multiplex immunofluorescence and tissue microarrays (198 HCC specimens), HLA‐DR⁺ tumor cells are identified as a distinctive feature of HBV^+^HCC. These tumor cells uniquely express MHC class II molecules, typically restricted to antigen‐presenting cells, and correlate with immune checkpoint activation and PD‐L1 expression, potentially contributing to an immunosuppressive microenvironment specific to HBV^+^HCC. Trajectory analysis revealed distinct CD8⁺ T‐cell differentiation pathways in HBV^+^HCC, characterized by enhanced exhaustion and stem‐like phenotypes. HLA‐DR⁺ tumor cells are associated with increased recruitment of CD8⁺ T cells and correlated with T‐cell exhaustion, potentially contributing to a suppressive TME. Clinically, high proportions of HLA‐DR⁺ tumor cells are linked to poor survival outcomes, especially when accompanied by elevated PD‐L1 expression, suggesting that HLA‐DR⁺ tumor cells may serve as a potential predictive biomarker for immunotherapy efficacy in HCC. Collectively, the findings highlight HLA‐DR⁺ tumor cells as a distinctive feature of HBV‐associated HCC (HBV^+^HCC), providing novel insights into possible immunosuppressive mechanisms and therapeutic targets for immunotherapy in this disease context.

## Graphical abstract

This study integrates scRNA‐seq, multiplex immunofluorescence, and clinical data to identify HLA‐DR⁺tumor cells uniquely enriched in Hepatocellular carcinoma (HCC) with hepatitis B virus (HBV). These cells express MHC II, associate with PD‐L1, recruit and exhaust CD8⁺T cells, predict poor survival, and serve as potential biomarkers for immunotherapy response.

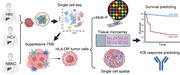

## 1 Introduction

Hepatocellular carcinoma (HCC) is a major global health concern and the most common form of primary liver cancer.^[^ ^1^ ^]^ Chronic hepatitis B virus (HBV) and hepatitis C virus (HCV) infections are among the most prevalent causes of HCC worldwide, particularly in regions with high endemicity.^[^ ^2^ ^]^ Additionally, non‐B non‐C (NBNC) HCC, which is increasingly linked to metabolic syndrome and nonalcoholic steatohepatitis, reflects the evolving etiological landscape of liver cancer.^[^ ^3^, ^4^ ^]^ Despite recent advances in early detection and targeted therapies, HCC remains highly lethal due to frequent recurrence, immune evasion, and the challenges of targeting a complex and heterogeneous tumor microenvironment (TME).

The TME of HCC exhibits significant heterogeneity that is strongly influenced by its underlying etiology. Recent advancements, particularly in single‐cell RNA sequencing (scRNA‐seq), have enabled unprecedented exploration of these differences, revealing substantial variation in immune composition and function across HBV‐, HCV‐, and NBNC‐associated HCC.^[^ ^5^, ^6^, ^7^ ^]^ Computational analyses have demonstrated marked heterogeneity in HCC, showing a robust correlation between HBV reads and cancer cell differentiation.^[^ ^8^ ^]^ Studies have explored HBV compartmentalization and differentiation within HCC, identifying HBV‐specific PD‐1^+^CD8^+^ tissue‐resident memory cells concentrated at tumor borders.^[^ ^9^ ^]^ These cells are closely linked to HBV‐induced hepatic damage and fibrosis in HCC patients. Despite these insights, no systematic investigation to date has fully leveraged scRNA‐seq to comprehensively compare the immune landscapes across HBV‐, HCV‐, and NBNC‐associated HCC. The existing literature often focuses on individual subtypes or provides limited mechanistic insights into how tumor‐intrinsic factors influence immune interactions. For example, the role of antigen presentation pathways, immune checkpoints, and tumor‐driven modulation of immune cells remains incompletely understood, particularly in HBV^+^HCC.^[^ ^5^, ^7^, ^10^, ^11^, ^12^ ^]^ These gaps in knowledge highlight the need for a systematic and integrated approach to uncover the immune and tumor‐intrinsic features that define the TME in HCC subtypes.

This study addresses these knowledge gaps by systematically analyzing the TME across HBV‐, HCV‐, and NBNC‐associated HCC using scRNA‐seq data from 160 samples representing 124 patients. Specifically, we identify HLA‐DR^+^ tumor cells as a hallmark feature of HBV^+^HCC. These tumor cells express major histocompatibility complex (MHC) class II molecules, which are typically restricted to professional antigen‐presenting cells. Our findings reveal that HLA‐DR^+^ tumor cells are not only enriched in HBV^+^HCC but are also associated with immune checkpoint activation, including PD‐L1 expression, and may contribute to the exhaustion of CD8^+^ T cells.

By systematically characterizing these differences and integrating transcriptomic, spatial, and clinical analyses, this study provides a comprehensive framework for understanding the immune landscapes of HCC subtypes. Moreover, our results highlight the potential of HLA‐DR^+^ tumor cells as prognostic biomarkers and therapeutic targets, particularly in the context of HBV^+^HCC. These findings lay the groundwork for future immunotherapeutic strategies tailored to the unique biology of HCC driven by distinct etiologies.

## 2 Result

### 2.1 HBV^+^HCC Demonstrates an Enhanced Immunosuppressive Environment

**Figure 1:**
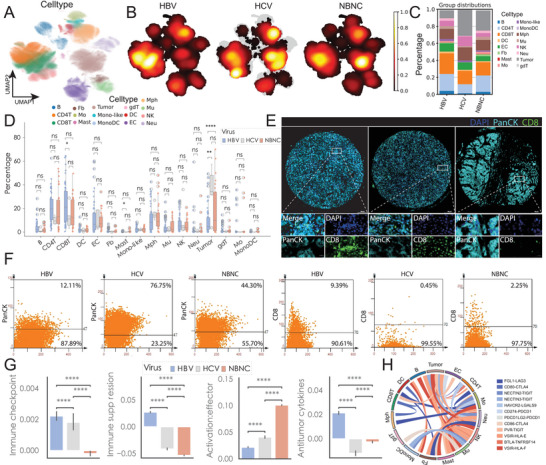
Characterization of the immune landscape in hepatitis B virus (HBV)‐, hepatitis C virus (HCV)‐ and non‐B non‐C (NBNC)‐associated hepatocellular carcinoma (HCC) (A) Uniform Manifold Approximation and Projection（UMAP） projection of single‐cell transcriptomic data from 160 samples across 124 HCC patients. Each point represents an individual cell, color‐coded by major cell types: B cells (B), fibroblasts (Fb), CD4⁺ T cells (CD4T), CD8⁺ T cells (CD8T), monocytes (Mo), monocyte‐derived dendritic cells (MonoDC), conventional dendritic cells (DC), endothelial cells (EC), mast cells (Mast), macrophages (Mph), neutrophils (Neu), NK cells (NK), and tumor epithelial cells (Tumor). (B) Density plots showing distribution cell populations in HBV+ (left), HCV+ (middle), and NBNC (right) samples. Warmer colors represent higher cell density within each viral etiology group. (C) Stacked bar charts showing the proportional abundance of each major cell type within HBV, HCV, and NBNC cohorts. Cell types are color‐coded as in panel (A). (D) Boxplots comparing the frequencies of each cell subset across HBV, HCV, and NBNC patient groups. Pairwise statistical comparisons were performed using the two‐sided Wilcoxon rank‐sum test. “ns” indicates non‐significance. (E) Representative multiplex immunofluorescence (mIF) images from tissue microarrays of HBV+ (left), HCV+ (middle), and NBNC (right) tumors. PanCK (cyan) marks epithelial tumor cells, CD8 (green) labels cytotoxic T cells, and DAPI (blue) stains nuclei. Insets show magnified tumor–immune interfaces. (F) Quantification of PanCK⁺ tumor and CD8⁺ T‐cell populations from mIF data. Scatterplots illustrate population proportions for HBV, HCV, and NBNC tumors, with percentages reflecting cell distribution in each group. (G) Bar graphs comparing expression of key immune regulatory features among HBV, HCV, and NBNC groups. Categories include immune checkpoint molecules, suppressive markers, activation/effector ratios, and antitumor cytokines. Pairwise statistical comparisons were performed using the Wilcoxon rank‐sum test; significance is indicated as ****p < 0.0001, ***p < 0.001, **p < 0.01, *p < 0.05. (H) Circos plot showing predicted ligand–receptor interactions among major cell types in the HBV+ HCC group. Chord thickness represents interaction strength.

Analysis of the immune microenvironment in liver cancer revealed significant heterogeneity across different viral infection contexts. Through public scRNA‐seq^[^ ^13^ ^]^ of 160 samples from 124 patients, we identified distinct cellular compositions among HBV, HCV, and NBNC groups. We categorized cell types into lymphocytes, myeloid cells, tumor cells, and others (**Figure** **1**A), with validation through marker gene expression (Figure S1A, Supporting Information). Clinical characteristics varied significantly among groups, with NBNC cases showing higher proportions of female patients, absence of cirrhosis, and more advanced‐stage cancers compared to HBV cases. Additionally, HBV‐associated samples demonstrated a higher prevalence of HCC compared to other cancer types (Figure S1B–I, Supporting Information).

Comprehensive transcriptomic analysis revealed distinct molecular profiles among HBV, HCV, and NBNC samples within individual cell types, highlighting the unique microenvironment associated with each viral context (Figure S2A, Supporting Information). Furthermore, HBV samples showed the highest proportion of lymphocytes, particularly CD8^+^ T cells, while NBNC samples exhibited the lowest lymphocyte proportion but higher tumor cell purity (Figure 1C,D). These findings were validated through tissue microarray (TMA) analysis and multicolor immunofluorescence staining of 198 liver cancer patient samples, confirming higher CD8+ T cell proportions in HBV^+^ samples compared to HCV^+^ and NBNC samples (Figure 1E,F; Figure S2B,C, Supporting Information). Conversely, NBNC samples demonstrated a higher proportion of PanCK^+^ tumor cells (Figure 1E,F; Figure S2B,C, Supporting Information).

Further immune status analyses revealed distinctive patterns among the groups. HBV+ samples exhibited paradoxical immune characteristics: the highest expression of immune checkpoint molecules and suppression markers, coupled with the lowest activation and effector signatures, yet maintaining the highest levels of antitumor cytokines (Figure 1G). In contrast, NBNC samples showed the lowest levels of these cytokines (Figure 1G). Cell–cell communication analysis using CellPhoneDB demonstrated enhanced immune checkpoint ligand‐receptor activation in HBV^+^ samples, particularly in tumor cell‐immune cell interactions, compared to HCV^+^ and NBNC groups (Figure 1H; Figure S2D, Supporting Information). These findings emphasize the heightened immunosuppressive environment associated with HBV infection in HCC.

### 2.2 HBV^+^HCC Cancer Cells Exhibit Elevated MHC Class II HLA‐DR Expression and Immune Modulatory Features

**Figure 2:**
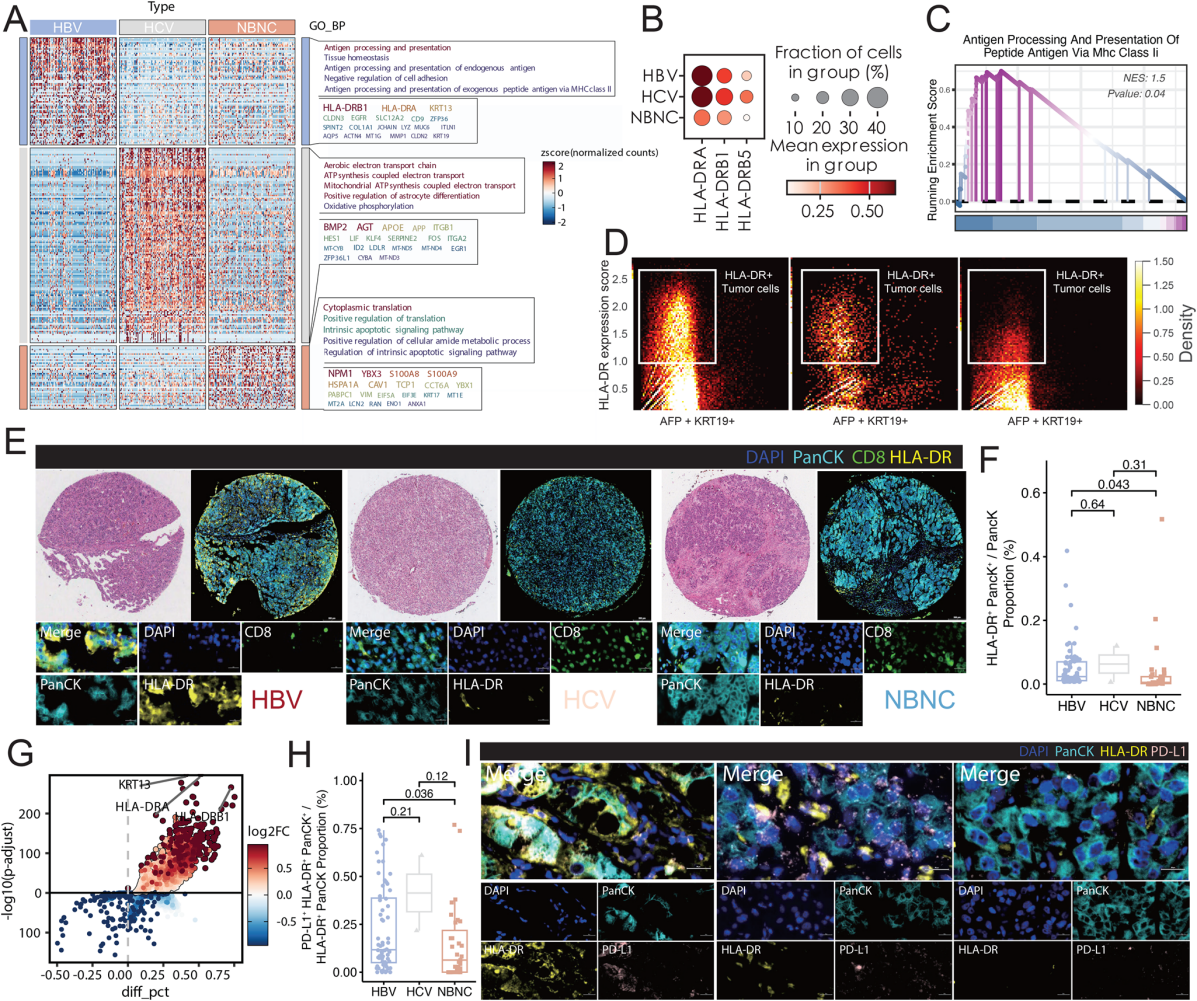
Enhanced HLA‐DR Expression in hepatitis B virus (HBV)‐Associated Tumors Compared to hepatitis C virus (HCV) and non‐B non‐C (NBNC) (A) Heatmap showing differentially expressed genes in tumor epithelial cells from HBV, HCV, and NBNC patient groups. Genes are grouped by Gene Ontology (GO) terms. Color intensity represents z‐scored normalized expression values; red indicates high expression and blue low expression. (B) Bubble plot summarizing expression of MHC class II genes (*HLA‐DRA*, *HLA‐DRB1*, *HLA‐DRB5*) across HBV, HCV, and NBNC tumor groups. Dot size indicates the proportion of cells expressing each gene; color scale reflects average expression within each group. (C) Gene Set Enrichment Analysis comparing HBV and NBNC tumor cells. Enrichment of the “Antigen Processing and Presentation via MHC Class II” pathway is significant in HBV tumors (Normalized Enrichment Score [NES] = 1.5, P = 0.04). (D) Density plots of *HLA‐DR* expression in tumor cells stratified by *AFP* and *KRT19* expression. HLA‐DR⁺ cells (top left quadrants) are more frequent in HBV tumors, particularly among AFP⁺KRT19⁺ double‐positive cells, compared to HCV and NBNC. (E) Multiplex immunofluorescence (mIF) images of representative tissue microarray cores from HBV (left), HCV (middle), and NBNC (right) patients. Panels show H&E staining and merged fluorescent channels: DAPI (nuclei, blue), PanCK (tumor marker, cyan), CD8 (green), and HLA‐DR (yellow). Insets highlight tumor regions with HLA‐DR⁺ tumor cells. (F) Quantification of HLA‐DR⁺PanCK⁺ tumor cells as a proportion of total PanCK⁺ cells across HBV, HCV, and NBNC groups. Pairwise comparisons were performed using the Wilcoxon rank‐sum test; exact P values are indicated above. (G) Volcano plot from scRNA‐seq comparing HLA‐DR⁺ versus HLA‐DR⁻ tumor cells. Genes significantly enriched in HLA‐DR⁺ cells (e.g., *HLA‐DRA*, *HLA‐DRB1*, *KRT13*) are shown in red; x‐axis = difference in cell fraction (diff_pct), y‐axis = ‐log10 adjusted P value. (H) Boxplot quantifying PD‐L1⁺HLA‐DR⁺PanCK⁺ tumor cells across HBV, HCV, and NBNC cohorts. Statistical comparisons were performed using Wilcoxon rank‐sum tests; P values are indicated. (I) High‐magnification mIF images showing co‐localization of HLA‐DR (yellow) and PD‐L1 (magenta) within PanCK⁺ tumor cells (cyan). DAPI (blue) marks nuclei. White arrows indicate HLA‐DR⁺PD‐L1⁺ double‐positive tumor cells, emphasizing immune evasion potential via antigen presentation and checkpoint signaling.

To investigate the potential mechanisms underlying immune and tumorigenic differences in HBV‐associated liver cancer, we isolated all cancer cells for further functional analysis. Compared to NBNC‐derived cancer cells, HBV‐derived cancer cells exhibited lower apoptosis, G2M checkpoint activity, and P53 signaling, but higher Myc and Kras pathway activation (Figure S3A, Supporting Information). Additionally, genomic instability analysis using inferred copy number variations (inferCNV) revealed that HBV cancer cells had lower copy number variation (CNV) scores compared to NBNC cancer cells (Figure S3B,C, Supporting Information). Leveraging the CNV score, we identified malignant cancer cells for further differential and pathway enrichment analyses. Surprisingly, differential expression analysis revealed that HBV cancer cells had significantly upregulated pathways associated with antigen processing and presentation (**Figure** **2**A). Specifically, MHC class II genes, including *HLA‐DRA*, *HLA‐DRB1*, and *HLA‐DRB5*, were highly expressed in HBV cancer cells, both in terms of expression levels and the proportion of cells expressing these genes (Figure 2A). These MHC class II genes, typically associated with antigen‐presenting cells such as dendritic cells and macrophages,^[^ ^14^, ^15^ ^]^ were not similarly elevated in NBNC‐derived cancer cells (Figure 2B). In contrast, no significant differences were observed in the expression of MHC class I genes across groups (Figure S3D, Supporting Information). Gene Set Enrichment Analysis (GSEA) further confirmed the activation of antigen presentation via the MHC class II pathway in HBV cancer cells (Figure 2C).

To quantify this finding, we developed an HLA‐DR score based on all upregulated HLA‐DR genes (*HLA‐DRA*, *HLA‐DRB1*, and *HLA‐DRB5*) and found that HBV cancer cells exhibited significantly higher HLA‐DR scores than NBNC cancer cells (Figure 2D). Validation at the protein level using multi‐IF on HCC TMAs confirmed that HBV samples had a higher proportion of PanCK^+^ HLA‐DR^+^ cells compared to NBNC samples (Figure 2E,F; Figure S4A,B, Supporting Information).

To explore the potential mechanisms behind the presence of HLA‐DR^+^ cancer cells and their relationship with the suppressive TME, we conducted differential analyses between high and low‐HLA‐DR‐expressing cancer cells. Volcano plot analysis highlighted that *HLA‐DRA* and *HLA‐DRB1* were significantly upregulated in high HLA‐DR cancer cells, particularly in HBV samples (Figure 2G). Further pathway analysis revealed enrichment of the PD‐L1 signaling pathway in high HLA‐DR‐expressing cells (Figure S4C, Supporting Information), aligning with our multi‐IF findings that HBV‐derived cancer cells also exhibited higher PD‐L1 expression compared to NBNC‐derived cells (Figure S4D, Supporting Information). These results were further validated by multi‐IF analysis, which demonstrated a significantly higher proportion of HLA‐DR^+^ PanCK^+^ cells, as well as HLA‐DR^+^ PD‐L1^+^ PanCK^+^ cells, in HBV samples compared to NBNC samples (Figure 2H,I; Figure S4E, Supporting Information, p < 0.05). This suggests a mechanistic link between elevated HLA‐DR expression in HBV cancer cells and the immunosuppressive TME mediated by PD‐L1. These findings highlight a novel role for HLA‐DR^+^ cancer cells in HBV‐associated liver cancer, linking antigen presentation pathways with immune checkpoint activation.

### 2.3 Distinct CD8^+^ T Cell Dynamics and Exhaustion Phenotypes in HBV^+^HCC

**Figure 3:**
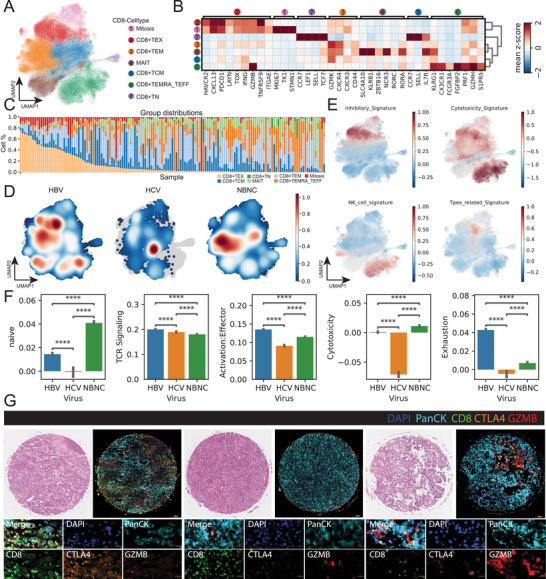
Single‐Cell Landscape of CD8+ T Cells in hepatitis B virus (HBV)‐, hepatitis C virus (HCV)‐, and non‐B non‐C (NBNC)‐Associated hepatocellular carcinoma (HCC) (A) Uniform Manifold Approximation and Projection (UMAP) projection of CD8⁺ T cells from HBV, HCV, and NBNC tumor samples, clustered by functional subsets. Colors indicate: naïve (CD8⁺ TN), central memory (CD8⁺ TCM), effector memory (CD8⁺ TEM), terminal effector (CD8⁺ TEMRA/TEFF), mitotic, exhausted (CD8⁺ TEX), and mucosal‐associated invariant T cells (MAIT). (B) Heatmap of signature gene expression across CD8⁺ T‐cell subsets. Each column represents a marker gene; rows are grouped by T‐cell subtype. Subset‐specific enrichment of inhibitory, cytotoxic, and memory‐related genes (e.g., *PDCD1*, *CTLA4*, *PRF1*, *ZNF683*) illustrates distinct transcriptional programs. (C) Stacked bar plot showing the relative distribution of CD8⁺ T‐cell subsets in each sample, stratified by etiology (HBV, HCV, NBNC). Substantial inter‐patient heterogeneity is evident within and across groups. (D) Subset density maps projected onto the CD8⁺ T‐cell UMAP space, shown separately for HBV (left), HCV (middle), and NBNC (right) samples. Warmer colors indicate higher density of localized subsets (e.g., CD8⁺ TEX or TEMRA cells). (E) UMAP feature plots showing functional module scores across all CD8⁺ T cells. Signature modules include: inhibitory (top left), cytotoxic (top right), NK‐like (bottom left), and Tpex‐related (stem‐like exhausted) (bottom right). Signature scores are color‐scaled (red = high, blue = low). (F) Bar graphs comparing functional pathway scores between HBV, HCV, and NBNC cohorts. Metrics include naïve phenotype, T cell receptor (TCR) signaling strength, activation/effector potential, cytotoxicity, and exhaustion levels. Statistical significance was assessed using Wilcoxon rank‐sum tests for all pairwise comparisons; ****p < 0.0001. (G) Representative multiplex immunofluorescence images of tissue microarray sections from HBV (left), HCV (middle), and NBNC (right) tumors. Panels show H&E (top row) and merged fluorescence channels (bottom row): DAPI (nuclei, blue), PanCK (tumor cells, cyan), CD8 (green), CTLA4 (orange), and GZMB (granzyme B, red). Insets highlight tumor–immune interfaces with co‐expression of CTLA4 and GZMB in CD8⁺ T cells.

To characterize the elevated CD8^+^ T cell population in HBV+HCC, we isolated all CD8^+^ T cells and confirmed these cells by using *CD3E* and *CD8A* markers (Figure S5A, Supporting Information). Through comprehensive re‐clustering analysis, we identified seven distinct CD8^+^ T cell subtypes: central memory (TCM), effector memory (TEM), TEM re‐expressing *CD45RA* and *CD27* (TEMRA_TEFF), naïve (TN), mucosal‐associated invariant T cells (MAIT), exhausted T cells (TEX), and mitotic exhausted T cells (**Figure** **3**A,B).

Our quantitative analysis revealed that TEM, TEMRA_TEFF, and TCM constituted the predominant subpopulations, with TEX, MAIT, mitotic cells, and TN present in smaller proportions (Figure S5B, Supporting Information). Notably, both TEX and mitotic exhausted cells demonstrated exhaustion characteristics, though mitotic cells uniquely expressed stem‐like markers (*CCR7*, *LEF1*, *SELL*) alongside exhaustion markers (Figure 3B; Figure S5D, Supporting Information). The distribution of these CD8^+^ T cell subpopulations varied significantly among patients, reflecting the heterogeneous nature of the immune microenvironment (Figure 3C). This heterogeneity strongly correlated with viral status: HBV^+^HCC patients showed enrichment of TEX, MAIT, and TEMRA_TEFF populations, while NBNC^+^HCC patients predominantly displayed TEM cells (Figure 3D; Figure S6A,B, Supporting Information). Functional characterization revealed that TEX and mitotic cells maintained cytotoxic capabilities despite expressing inhibitory markers, with TEX cells specifically enriched for the Tpex signature – a crucial determinant of immunotherapy response ^[^ ^16^ ^]^ (Figure 3E). HBV^+^ patient‐derived CD8*^+^* T cells exhibited enhanced activation, effector function, exhaustion, T cell receptor (TCR) signaling, and cytotoxicity signatures while showing reduced naïve‐like characteristics (Figure 3F). TMA analysis confirmed these findings, demonstrating elevated *CTLA4* expression but diminished *GZMB* levels in HBV^+^ CD8^+^ T cells compared to NBNC^+^HCC counterparts (Figure 3G; Figures S6C, Supporting Information).

RNA velocity analysis revealed distinct CD8⁺ T‐cell differentiation trajectories across viral etiologies. In HBV‐associated HCC(HBV^+^HCC), we observed a sustained enrichment of both terminally exhausted (TEX) and mitotic CD8⁺ T cells at the endpoint of pseudotime progression (Figure S6D, Supporting Information). These exhausted populations predominantly arose from TCM and early effector subsets, indicating a gradual and persistent transition toward dysfunction under chronic antigen exposure. Notably, mitotic cells in HBV+ HCC exhibited a biphasic pattern, with an initial peak during early pseudotime followed by a decline and a second resurgence at later stages—suggesting potential proliferative bursts within the exhausted pool. In contrast, HCV‐associated HCC displayed an earlier onset of T‐cell exhaustion, followed by re‐engagement of mitotic subsets and eventual accumulation of TEX cells (Figure S6E, Supporting Information). This pattern may reflect dynamic reactivation of proliferative intermediates under ongoing antigenic pressure. These divergent trajectories underscore the unique immunological context of HBV infection, characterized by a stable expansion of exhausted and stem‐like CD8⁺ T cells, with implications for therapeutic strategies targeting T‐cell reinvigoration in HBV+ HCC.

### 2.4 Impact of HLA‐DR^+^ Tumor Cells on the TME and CD8^+^ T‐Cell Dynamics and Exhaustion Phenotypes

**Figure 4:**
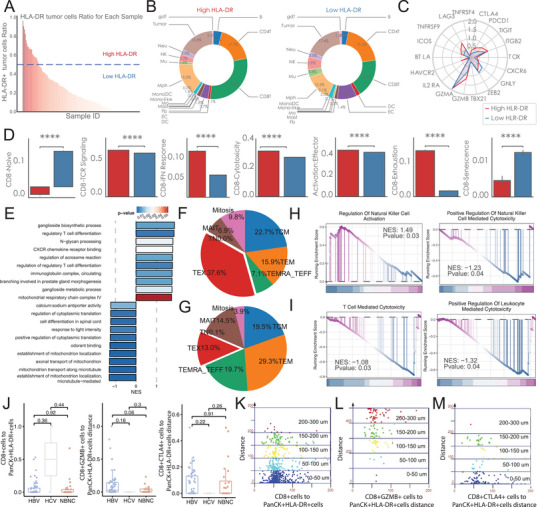
Impact of HLA‐DR^+^ Tumor Cells on the Tumor Microenvironment and CD8^+^ T‐Cell Dynamics (A) Bar plot showing the distribution of HLA‐DR⁺ PanCK⁺ tumor‐cell ratios across all patient samples. A median cut‐off (dashed line) defines “High HLA‐DR” and “Low HLA‐DR” tumor groups for subsequent analyses. (B) Pie charts depicting immune cell composition in tumors with High (left) versus Low (right) HLA‐DR expression. High HLA‐DR tumors exhibit increased proportions of CD8⁺ T cells and macrophages (Mph), with relative reductions in fibroblasts (Fb), monocytes (Mo), and other subsets. (C) Radar plot comparing average expression of immunoregulatory and cytotoxic genes (e.g., *LAG3*, *TNFRSF9*, *CTLA4*, *PDCD1*, *TIGIT*, *CXCR6*, *GZMB*, *TBX21*) in CD8⁺ T cells from High versus Low HLA‐DR tumors. Most inhibitory and cytotoxic markers are elevated in the High HLA‐DR group. (D) Bar graphs quantifying CD8⁺ T‐cell functional states—including naïve, T cell receptor (TCR) signaling, interferon response, cytotoxicity, activation, exhaustion, and senescence—in High versus Low HLA‐DR tumors. Wilcoxon rank‐sum tests show significant increases in activation, exhaustion, and cytotoxic signatures in the High HLA‐DR group (****p < 0.0001). (E) Gene Ontology (GO) pathway enrichment analysis comparing tumors with High versus Low HLA‐DR expression. Enriched pathways in the High group include regulatory T‐cell differentiation and mitochondrial respiration processes. (F, G) Pie charts showing the distribution of CD8⁺ T‐cell subsets in High (F) and Low (G) HLA‐DR tumors. High HLA‐DR tumors are enriched in exhausted cells (TEX) and mitotic populations, while Low HLA‐DR tumors have more central memory cells (TCM) and terminal effector cells (TEMRA/TEFF) cells. (H, I) Gene Set Enrichment Analysis (GSEA) of TEX cells reveals upregulated cytotoxic‐related signatures (e.g., NK activation, T‐cell cytolytic activity) in High HLA‐DR tumors versus Low. Normalized enrichment scores (NES) and P values are shown. (J) Box plots comparing spatial distances between CD8⁺ T cells and HLA‐DR⁺ tumor cells across hepatitis B virus (HBV), hepatitis C virus (HCV), and non‐B non‐C (NBNC) groups. No significant differences were observed between viral etiologies, suggesting that proximity alone does not fully explain functional differences. Wilcoxon rank‐sum tests used for pairwise comparisons. (K–M) Scatter plots showing the relationship between CD8⁺ T‐cell functional phenotype (CD8⁺, GZMB⁺, CTLA4⁺) and distance to HLA‐DR⁺ tumor cells. Shorter distances correlate with higher CTLA4 expression and reduced GZMB, indicating spatially associated immune exhaustion.

To investigate whether HLA‐DR^+^ tumor cells influence the TME, we stratified patients into two groups based on the proportion of HLA‐DR^+^ cancer cells: high HLA‐DR tumors versus low HLA‐DR tumors (**Figure** **4**A). Tumors with higher HLA‐DR^+^ cancer cell proportion contained more CD8^+^ T cells and macrophages (Figure 4B). Further analyses showed that these high HLA‐DR^+^ cancer cell tumors exhibited elevated levels of *LAG3*, *TNFRSF9*, *CTLA4*, *PDCD1*, *TIGIT*, *ITGB2*, and *GZMB* (Figure 4C).

We next examined the impact of HLA‐DR^+^ cancer cells on CD8^+^ T‐cell subtypes (Figure S7A, Supporting Information). Notably, CD8‐naïve T cells in high HLA‐DR tumors expressed more ITGB2, while the TEX (exhausted) and mitotic subsets were most affected. TEX cells from high HLA‐DR tumors had increased *LAG3*, *PDCD1*, *HAVCR2*, and *GZMB*, and mitotic cells showed higher *LAG3*, *CTLA4*, *PDCD1*, *TIGIT*, *ITGB2*, *HAVCR2*, and *GZMB*. Functional signatures confirmed that CD8^+^ T cells in high HLA‐DR tumors had elevated TCR signaling, interferon responses, cytotoxicity, exhaustion, and activation, with lower naïve and senescence signatures (Figure 4D). Although this pattern held true across different viral statuses, the upregulated TCR signaling was most pronounced in HBV^+^HCC (Figure S7B, Supporting Information). Additional pathway enrichment revealed that CD8^+^ T cells in high HLA‐DR tumors also had increased regulatory T‐cell differentiation pathways (Figure 4E). Consistent with these observations, high HLA‐DR tumors showed enrichment of TEX, mitotic exhausted T cells, and TCM, while low HLA‐DR tumors had more TEFF, MAIT, and TEM (Figure 4F,G). Separate GSEA analyses within each CD8^+^ T‐cell subset indicated that TEX cells from high HLA‐DR tumors displayed greater cytotoxic potential than their counterparts in low HLA‐DR tumors (Figure 4H–I).

To validate these findings, we performed multi‐IF and measured the spatial distribution of CD8^+^ T cells relative to PanCK^+^ HLA‐DR^+^ tumor cells in HBV^+^HCC. We found no significant differences in the distance between PanCK^+^ HLA‐DR^+^ cells and various CD8^+^ T‐cell subsets (including CD8^+^GZMB^+^ and CD8^+^CTLA4^+^ cells) across HBV, HCV, and NBNC groups (Figure 4J). This suggests that the proportion of HLA‐DR+ tumor cells, rather than virus status, plays a key role in shaping the TME. Further distance‐based analyses around HLA‐DR^+^ tumor cells revealed that closer proximity correlated with a higher density of CD8^+^ T cells. However, these nearby CD8^+^ T cells were more likely to express CTLA4 and less likely to express GZMB (Figure 4K–M). These findings are consistent with the high HLA‐DR phenotype observed in HBV tumors, in which tumor cells appear to recruit CD8^+^ T cells but induce a more exhausted phenotype characterized by diminished *GZMB* expression.

Taken together, our data suggest that HLA‐DR^+^ tumor cells may attract CD8^+^ T cells while simultaneously driving their exhaustion, thereby contributing to an immunosuppressive TME. Although direct interactions likely play a central role, we cannot exclude contributions from additional cytokines, cell types, or other TME factors that may further modulate these interactions.

### 2.5 Clinical Implications and Prognostic Value of HLA‐DR^+^ Tumor Cells in HCC

To establish the clinical significance of our findings, we retrospectively analyzed 729 patients with HCC treated at Henan Provincial Hospital between 2020 and 2025 (Table S1, Supporting Information). Survival analyses showed no significant differences among patients with HBV^+^ HCC, HCV^+^ HCC, or NBNC HCC in overall survival (OS, *p* = 0.15) or progression‐free survival (PFS, *p* = 0.37) (Figure S8A, Supporting Information). These results were corroborated by OS data from the TCGA‐LIHC cohort (*p* = 0.35) (Figure S8A, Supporting Information). Thus, viral status alone was not predictive of patient outcomes, indicating the need for additional prognostic markers.

**Figure 5:**
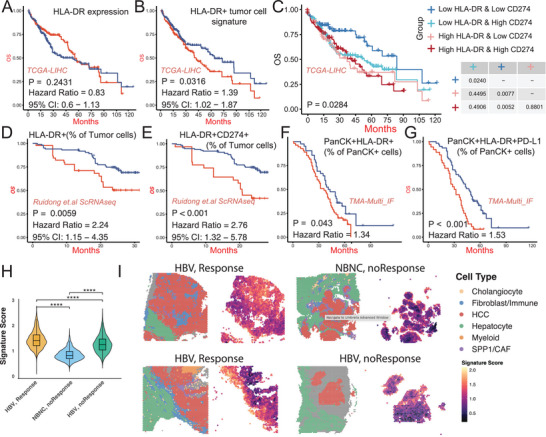
Clinical Implications and Prognostic Value of HLA‐DR^+^ Tumor Cells in Hepatocellular Carcinoma (HCC). (A) Kaplan–Meier survival curve from the TCGA‐LIHC cohort comparing overall survival (OS) in patients with high (red) versus low (blue) total HLA‐DR gene expression. No significant difference was observed (P = 0.2431; HR = 0.83; 95% CI: 0.60–1.13). (B) OS analysis based on a HLA‐DR⁺ tumor‐cell signature derived from scRNA‐seq data. Patients with high signature scores (red) exhibited significantly worse prognosis (P = 0.0316; HR = 1.39; 95% CI: 1.02–1.87). (C) Combined stratification using HLA‐DR⁺ tumor‐cell signature and *CD274* (PD‐L1) expression levels further refines prognostic prediction. Patients were divided into four groups: low HLA‐DR/low CD274 (light blue), low HLA‐DR/high CD274 (blue), high HLA‐DR/low CD274 (pink), and high HLA‐DR/high CD274 (red). Log‐rank test indicates significant separation of survival curves (P = 0.0284), with worst outcomes in the high HLA‐DR + high CD274 group. (D–E) Independent validation using scRNA‐seq data with clinical outcome information (Ruidong et al.). Kaplan–Meier curves show that patients with higher proportions of (D) HLA‐DR⁺ tumor cells (P = 0.0059; HR = 2.24; 95% CI: 1.15–4.35) and (E) HLA‐DR⁺CD274⁺ tumor cells (*p* < 0.001; HR = 2.76; 95% CI: 1.32–5.78) have significantly reduced OS. (F–G) Protein‐level validation using multiplex immunofluorescence of tissue microarrays (n = 98 HCC cases). Kaplan–Meier survival curves based on the proportion of PanCK⁺HLA‐DR⁺ tumor cells (F; P = 0.043; HR = 1.34) and PanCK⁺HLA‐DR⁺CD274⁺ tumor cells (G; *p* < 0.001; HR = 1.53). Patients with high double‐positive tumor burden exhibit the shortest OS. (H) Violin plots comparing HLA‐DR signature scores across three patient groups: hepatitis B virus (HBV)^+^ HCC responders (orange), HBV⁺ non‐responders (green), and non‐B non‐C (NBNC) non‐responders (blue) to anti‐PD‐1 therapy. HBV responders show significantly higher HLA‐DR signature scores. Wilcoxon rank‐sum test used for pairwise comparisons (****p < 0.0001). (I) Spatial transcriptomics maps displaying cell‐type annotations (left) and tumor‐specific HLA‐DR signature expression (right, magenta scale) in representative tumors. HBV responders show enriched HLA‐DR signature in tumor epithelial regions, whereas non‐responders (HBV, NBNC) show reduced expression, suggesting predictive value for immunotherapy response.

We next evaluated the prognostic utility of total HLA‐DR expression in the TCGA‐LIHC dataset. No survival difference emerged between patients with high versus low HLA‐DR expression (hazard ratio [HR] = 1.12, *p* = 0.243; **Figure** **5**A), likely due to confounding by HLA‐DR^+^ antigen‐presenting cells in the TME. To address this limitation, we developed and validated a tumor‐specific HLA‐DR signature from scRNA‐seq data, focusing on malignant cells rather than the broader stromal compartment. Notably, patients with a high HLA‐DR^+^ tumor cell signature exhibited significantly worse OS (HR = 1.39, 95% confidence interval [CI]: 1.03–1.87, *p* = 0.032; Figure 5B). Integrating *CD274* (PD‐L1) expression enhanced prognostic stratification, with patients demonstrating low expression of the HLA‐DR^+^ tumor cell signature and low CD274 enjoying the most favorable survival outcomes (*p* = 0.028; Figure 5C).

To further validate these observations, we leveraged the scRNA‐seq dataset from Ruidong et al.,^[^ ^13^ ^]^ which included matched survival information. Patients harboring elevated proportions of HLA‐DR^+^ tumor cells (HR = 2.24, 95% CI: 1.15–4.35, *p* = 0.006) or HLA‐DR^+^CD274^+^ tumor cells (HR = 2.76, 95% CI: 1.32–5.78, *p* < 0.001) had significantly worse OS (Figure 5D,E). Finally, a protein‐level analysis using multiplex immunofluorescence (mIF) on a TMA of 198 HCC specimens—with matched survival data—corroborated these findings. Patients with high proportions of HLA‐DR^+^ tumor cells exhibited poorer outcomes (HR = 1.34, 95% CI: 1.01–1.78, *p* = 0.043; Figure 5F), and this effect was even more pronounced when considering HLA‐DR^+^CD274^+^ double‐positive tumor cells (HR = 1.53, 95% CI: 1.24–1.89, *p* < 0.001; Figure 5G). Collectively, these data underscore the importance of tumor‐specific HLA‐DR expression, particularly in conjunction with CD274 status, as a robust prognostic biomarker in HCC.

### 2.6 HLA‐DR^+^ Tumor Cells May can as Predictive Markers for Immunotherapy

To evaluate the potential clinical utility of HLA‐DR^+^ tumor cells in immunotherapy response prediction, we analyzed a cohort of HCC patients treated at Henan Provincial Hospital between 2020 and 2025. These patients were treated with either anti‐PD‐1 immunotherapy alone or a combination of immunotherapy and targeted therapy. Objective response rates were significantly higher in HBV^+^HCC patients compared to HCV^+^HCC and NBNC patients (Figure S8B,C, Supporting Information).

To mechanistically understand this differential response, we leveraged a spatial transcriptomics dataset ^[^ ^17^ ^]^ comprising HCC specimens with matched immunotherapy outcomes. Quantitative analysis of tumor‐specific HLA‐DR expression revealed that HBV^+^HCC responders exhibited significantly elevated HLA‐DR levels (mean expression score: 2.8 ± 0.4) compared to both HBV^+^ nonresponders (1.4 ± 0.3, *p *< 0.001) and NBNC patients (1.1 ± 0.2, *p *< 0.001) (Figure 5H,I).

These findings establish HLA‐DR^+^ tumor cells as both a prognostic indicator and a potential predictive biomarker for immunotherapy efficacy in HCC. The pronounced association between HLA‐DR expression and treatment response in HBV^+^HCC suggests a mechanistic link between viral etiology, antigen presentation, and immunotherapy outcomes. These results provide a rational basis for prospective validation studies and the development of HLA‐DR‐guided patient stratification strategies for immunotherapy in HCC.

## 3 Discussion

This study provides a comprehensive analysis of the immune microenvironment and tumor‐intrinsic features of HCC under different viral infection contexts. By leveraging scRNA‐seq, we revealed the distinct immune profiles associated with HBV, HCV, and NBNC etiologies. Notably, HBV^+^HCC demonstrated a heightened immunosuppressive environment characterized by an increased infiltration of CD8^+^ T cells and elevated expression of immune checkpoint molecules. These findings underscore the critical role of HBV infection in reshaping the TME to favor immune evasion and tumor progression. 

One of the key discoveries was the identification of HLA‐DR^+^ tumor cells as a hallmark of HBV^+^HCC. These cells were associated with the activation of antigen presentation pathways and immune checkpoint signaling, particularly PD‐L1 expression. The presence of HLA‐DR^+^ tumor cells raises several hypotheses regarding their origin and function in the immunosuppressive TME. It is plausible that HBV infection directly induces the expression of MHC class II genes through viral‐mediated modulation of tumor cell epigenetics or transcriptional machinery. The chronic inflammatory state induced by HBV may further drive this expression via cytokine‐mediated signaling, particularly through interferon‐gamma,^[^ ^18^, ^19^, ^20^, ^21^ ^]^ which is known to upregulate MHC class II molecules in various cell types.^[^ ^22^, ^23^, ^24^ ^]^

The functional role of HLA‐DR+ tumor cells in the immunosuppressive environment is multifaceted.^[^ ^25^ ^]^ By presenting tumor antigens, these cells might paradoxically impair effective anti‐tumor immunity by inducing T‐cell exhaustion.^[^ ^26^ ^]^ Elevated PD‐L1 expression on HLA‐DR^+^ cells suggests that these cells actively engage immune checkpoints to suppress cytotoxic T‐cell activity.^[^ ^27^ ^]^ Moreover, the recruitment of CD8^+^ T cells by HLA‐DR^+^ tumor cells, followed by their subsequent exhaustion, highlights a potential mechanism by which these cells contribute to immune evasion. This process may also enhance tumor cell survival and proliferation within the hostile immune landscape. 

Our trajectory analysis of CD8^+^ T cells revealed distinct differentiation dynamics in HBV^+^HCC, characterized by an enrichment of TEX and mitotic subsets. This suggests a prolonged and chronic stimulation of the immune system in the context of HBV infection, which aligns with the hypothesis that HLA‐DR^+^ tumor cells exacerbate immune exhaustion. The stem‐like properties observed in TEX cells in HBV^+^HCC further suggest potential responsiveness to immune checkpoint inhibitors (ICIs. Indeed, we found that the co‐expression of HLA‐DR and PD‐L1 in tumor cells may serve as a predictive biomarker for ICI efficacy. 

Interestingly, HLA‐DR^+^ tumor cells may also influence the TME beyond direct T‐cell interactions. For example, these cells could secrete immunosuppressive cytokines or interact with other immune cell types, such as regulatory T cells or myeloid‐derived suppressor cells, to further dampen anti‐tumor immunity.^[^ ^28^, ^29^ ^]^ Future studies exploring these interactions could provide critical insights into the broader immunosuppressive mechanisms of HLA‐DR^+^ tumor cells. Our findings align with prior studies that emphasize the role of immune checkpoint signaling in HCC pathogenesis. Previous work has demonstrated that HBV infection enhances PD‐L1 expression on both immune and tumor cells, thereby facilitating immune evasion.^[^ ^3^, ^8^, ^30^, ^31^, ^32^ ^]^ However, this study provides a novel perspective by identifying the specific contribution of HLA‐DR^+^ tumor cells to this process. The observed upregulation of MHC class II genes in tumor cells, a phenomenon traditionally associated with antigen‐presenting cells, is consistent with emerging evidence in esophageal adenocarcinoma and lung cancer,^[^ ^28^, ^29^ ^]^ but its role in HBV^+^HCC has not been previously characterized. In contrast, NBNC‐associated HCC exhibited a distinct immune profile with reduced lymphocyte infiltration and higher tumor cell purity, consistent with the notion that metabolic‐driven HCCs often lack significant immune activation.^[^ ^33^, ^34^, ^35^, ^36^, ^37^ ^]^ This underscores the heterogeneity of the TME across different etiologies and highlights the need for tailored therapeutic approaches.

Despite the strengths of this study, several limitations warrant consideration. First, while scRNA‐seq provided high‐resolution insights into cellular heterogeneity, its reliance on dissociated cells may result in the loss of spatial context. Future studies should incorporate spatial transcriptomics to validate the spatial organization and interactions between HLA‐DR^+^ tumor cells and CD8^+^ T cells within the TME. Additionally, the regulatory mechanisms driving HLA‐DR expression in tumor cells remain unclear. Investigating potential epigenetic and transcriptional regulators, as well as the influence of HBV viral proteins, could uncover novel therapeutic targets. Furthermore, while we demonstrated the prognostic value of HLA‐DR^+^ tumor cells and their association with immune suppression, prospective clinical studies are needed to validate these findings and assess their utility in guiding immunotherapy. Combination therapies targeting both HLA‐DR‐mediated antigen presentation pathways and immune checkpoint signaling represent a promising avenue for future research. Finally, we acknowledge that our current findings are primarily based on integrative transcriptomic and histological analyses, and do not include direct functional assays to confirm the causal role of HLA‐DR⁺ tumor cells in driving CD8⁺ T‐cell exhaustion. However, the convergence of scRNA‐seq, pseudotime trajectory, and mIF data provides robust, multi‐layered evidence for this association. Notably, we observed co‐localization of HLA‐DR⁺ tumor cells with exhausted CD8⁺ T‐cell subsets, along with upregulation of immune checkpoint ligands such as PD‐L1, and a clear link to adverse clinical outcomes. These findings offer a compelling biological narrative that justifies the prioritization of HLA‐DR⁺ cells as therapeutic targets. We view this study as a foundational resource that establishes a strong rationale for future mechanistic investigations, including in vitro co‐culture systems and in vivo functional assays, to further elucidate the immunoregulatory functions of HLA‐DR⁺ tumor cells in HBV^+^HCC.

## 4 Conclusion

From a theoretical perspective, this study advances our understanding of the immunological heterogeneity of HCC and the role of tumor‐intrinsic factors in shaping the TME. By highlighting the interplay between HLA‐DR^+^ tumor cells and immune exhaustion, this study contributes to the growing body of evidence supporting the bidirectional communication between tumor and immune cells in cancer progression. From a clinical perspective, the identification of HLA‐DR^+^ tumor cells as a potential biomarker has significant prognostic and therapeutic implications. Patients with high HLA‐DR^+^ tumor cell signatures exhibited worse survival outcomes, and this effect was further amplified when combined with PD‐L1 expression. These findings suggest that HLA‐DR^+^ tumor cells could serve as predictive markers for immunotherapy responsiveness, particularly in HBV^+^HCC. 

In summary, this study provides a detailed characterization of the TME in HBV^+^HCC, with a focus on the role of HLA‐DR^+^ tumor cells in promoting immune suppression. By integrating transcriptomic, spatial, and clinical analyses, we uncovered novel mechanisms underlying immune evasion and highlighted the potential of HLA‐DR^+^ tumor cells as therapeutic targets and prognostic markers. These findings emphasize the need for etiology‐specific therapeutic strategies in HCC and underscore the importance of understanding tumor‐immune interactions in developing effective immunotherapies. By addressing a key gap in the field and providing actionable insights, this study lays the foundation for future research aimed at improving outcomes for patients with HCC, particularly those with HBV^+^HCC. 

## 5 Experimental Section

### Patient Cohort and Sample Collection

This study included a cohort of 729 HCC patients diagnosed at Henan Provincial Hospital between January 2020 and January 2025. All patients provided written informed consent, and the study protocol was approved by the Institutional Review Board of Henan Provincial Hospital. Demographic, clinical, and survival data were systematically collected through medical records and follow‐up visits. Patients were stratified into three etiological subgroups based on viral infection status: HBV‐positive, HCV‐positive, and NBNC. Stratification was determined through serological testing (HBsAg, anti‐HCV) and clinical history.

Formalin‐fixed paraffin‐embedded TMA samples were purchased from Shanghai Xinchao Biotechnology Co., Ltd. The TMA dataset consisted of 198 paired HCC and adjacent non‐tumor liver tissue samples. These samples were obtained from patients with known viral status and long‐term follow‐up data for survival analysis. Tissue collection was performed via surgical resection or core needle biopsy, followed by fixation in 10% neutral‐buffered formalin for 24–48 h, and paraffin embedding using standard histopathological protocols. Sections (4 µm) were used for immunofluorescence analyses following standard deparaffinization and antigen retrieval procedures.

### scRNA‐seq Library Preparation and Sequencing

scRNA‐seq data analyzed in this study were derived from the publicly available dataset reported by Ruidong et al.,^[^ ^13^ ^]^ which has been deposited in the Genome Sequence Archive at the National Genomics Data Center (Beijing, China) under BioProject ID PRJCA007744. The dataset includes 160 tumor and adjacent tissue samples derived from 124 patients with HBV‐, HCV‐, or NBNC‐associated HCC, with clinical metadata including sex, age, and disease etiology. Raw sequencing data were processed using Cell Ranger (version 6.0.0, 10x Genomics). The pipeline included demultiplexing, alignment to the GRCh38 human reference genome, and generation of gene‐barcode matrices.

### Single‐Cell Variational Inference (scVI)

scVI,(version 0.6.8) was employed for advanced processing and analysis of scRNA‐seq.^[^ ^38^ ^]^ scVI leverages deep learning combined with probabilistic modeling to effectively capture latent phenotypic features across the entire dataset. This method enables the reduction of high‐dimensional single‐cell data into a multidimensional latent representation, preserving essential cellular characteristics while mitigating technical noise and batch effects. The scVI pipeline involves training a variational autoencoder to model the latent space of cells based on their gene expression profiles. The resulting latent representations were then projected into a 2D space for visualization and downstream analysis. This dimensionality reduction was crucial for identifying and interpreting distinct cellular phenotypes and their relationships within the TME. Additionally, batch correction was performed intrinsically by scVI to ensure the integrity of cross‐sample comparisons. The scVI model was trained with default parameters, including a 128‐D latent space and a learning rate of 0.001. After training, cells were visualized using Uniform Manifold Approximation and Projection (UMAP) to create interpretable 2D projections. These low‐dimensional embeddings provided a comprehensive view of cell population structure, facilitating further clustering, trajectory, and cell‐cell interaction analyses.

### Preprocessing and Quality Control

Raw sequencing data were processed using Cell Ranger (v6.0, 10x Genomics) and aligned to the GRCh38 reference genome. Low‐quality cells (genes <200, mitochondrial gene content >10%) were excluded. DoubletFinder (v2.0.3) was used to remove cell doublets. Normalization, clustering, and differential expression analysis were performed using Seurat (v4.0) in R. Highly variable genes were identified using FindVariableFeatures (top 2000 genes). Data were scaled with ScaleData and principal component analysis (PCA) was performed. The first 20 principal components were used for graph‐based clustering via FindNeighbors and FindClusters (Louvain algorithm). UMAP was used for visualization.

### Cell Type Annotation and Subclustering

Clusters were annotated using marker genes validated in the literature. Differentially expressed genes were identified using FindAllMarkers (Wilcoxon rank‐sum test, Bonferroni correction). For sub‐clustering, the batch correction was applied using RunFastMNN, and clusters were refined through hierarchical clustering.

### Copy Number Variation Analysis (inferCNV)

To assess genomic instability, inferCNV (v1.8.1) was applied to scRNA‐seq data using stromal and immune cells as reference. CNV scores were computed, and heat maps visualizing chromosome‐wide alterations were generated. The significance of CNV differences among HBV, HCV, and NBNC groups was assessed using ANOVA.

### Trajectory Analysis of CD8^+^ T Cells

Cell trajectory inference was performed using scVelo (v0.2.4) to study transcriptional dynamics in CD8+ T‐cell subsets.^[^ ^39^ ^]^ Preprocessing included filtering and normalizing expression data (scv.pp.filter_and_normalize), computing first‐order moments (scv.pp.moments), and estimating RNA velocity (scv.tl.velocity). Latent time analysis (scv.tl.recover_latent_time) was performed to infer differentiation trajectories, and results were visualized using scv.pl.velocity_pseudotime.

### Cell‐Cell Communication Analysis

Cell–cell interactions or ligand‐receptor interactions within the TME were analyzed using CellPhoneDB (v2.1.7).^[^ ^40^ ^]^ Input files for statistical analysis included the raw count matrix extracted from Seurat objects and an annotation file specifying cell types. The “heatmap_plot” function of CellPhoneDB, along with the Circlize package (version 0.4.14), was employed to visualize the frequency of interactions between two cell subpopulations. The interaction strength potential between ligands and receptors, predicted based on their average expression levels, was visualized using the Circlize package (version 1.0.12). Significant ligand‐receptor pairs (*p* < 0.01) were extracted and presented. We conducted ligand‒receptor (LR) network analysis using CellPhoneDB with the following parameters: ′*–iterations = 1000—subsampling –subsampling‐log false—subsamplingnum‐ cells 1000–threshold 0.01.″* Predicted LR pairs with *p* < 0.01 were selected. To facilitate comparisons between datasets, LR mean intensities were normalized. The normalization was calculated as the percentage of the total intensity of predicted significant interacting pairs for ligands and receptors within each dataset, with a *p* value less than 0.01, and multiplied by 10^4^, representing the average intensity for every 10^4^ total mean intensities. LR pairs with no annotated receptor were filtered out.

### MIF

Paraffin‐embedded TMAs containing 198 HCC samples were stained using the Opal seven‐color multiplex immunohistochemistry kit (Akoya Biosciences). The primary antibodies used in this study were as follows:

- **Anti‐CD8** (1:200, BSM‐60235R, Bioss Antibodies): Alexa Fluor 520, incubation time: 5 min.
- **Anti‐CTLA‐4** (1:200, ZM‐0035, Zhongshan Golden Bridge Biotechnology): Alexa Fluor 570, incubation time: 5 min.
- **Anti‐GZMB** (1:100, ab255598, Abcam): Alexa Fluor 620, incubation time: 3 min.
- **Anti‐PD‐L1** (1:100, ab214572, Abcam): Alexa Fluor 700, incubation time: 10 min.
- **Anti‐HLA‐DR** (1:50, ab927, BioLegend): Alexa Fluor 660, incubation time: 15 min.
- **Anti‐PanCK** (1:200, hsm2431R, Bioss Antibodies): Alexa Fluor 690, incubation time: 10 min.

Following deparaffinization, antigen retrieval was conducted using citrate buffer (pH 6.0) at 95 °C for 30 min. Fluorescence signals were amplified using Opal fluorophores. Finally, sections were counterstained with DAPI and mounted using ProLong Gold Antifade Reagent (Invitrogen).

### Image Acquisition and Analysis

All stained slides were scanned at 20× magnification using the TissueFAXS platform (TissueGnostics, Vienna, Austria), which captured fluorescence spectra at 20 nm wavelength intervals from 420 to 720 nm with the same exposure time. These scan slides were used to extract the spectral characteristics of each fluorophore, establishing a spectral library used for the decomposition of multiplexed images using StrataQuest software (TissueGnostics, Vienna, Austria) for further analysis. Positive cutoff values were determined based on staining patterns and intensities for each primary antibody across all images. Spatial colocalization analysis of multiplexed imaging data was performed to reconstruct entire slides without introducing edge effects or data loss. StrataQuest software (TissueGnostics, Vienna, Austria) was used to calculate distances between cells of interest and the number of mutual neighbors between phenotypically distinct cells (maximum distance of 25 µm). The spatial distribution of CD8^+^ subsets was analyzed by measuring the distance of CD8^+^ CTLA4^+^ and CD8^+^ GZMB^+^ cells from PanCK^+^ HLA‐DR^+^ tumor cells.

### Differential Expression and GSEA

Differential expression analysis was performed using Seurat's FindMarkers function (Wilcoxon rank‐sum test, adjusted *p* < 0.05). Gene Ontology (GO) and Kyoto Encyclopedia of Genes and Genomes (KEGG) enrichment analyses were conducted using clusterProfiler (v3.0.4). Gene Set Variation Analysis (GSVA) (v1.14.1) was used to assess T‐cell‐related signatures.

### Survival Analysis

Kaplan‐Meier survival curves were generated using the survival (v2.42‐3) and survminer (v0.4.9) packages in R. Comparisons were made using the log‐rank test. Cox proportional hazard regression models were constructed to assess the impact of HLA‐DR tumor cell signatures on survival. Patients were stratified by high versus low HLA‐DR expression, and HRs were reported.

### Spatial Transcriptomics Data Acquisition and Processing

To investigate the spatial immunological architecture of HCC in relation to immunotherapy response, we re‐analyzed publicly available spatial transcriptomics datasets originally generated by Liu et al.^[^ ^17^ ^]^ These data, which include annotated immunotherapy outcomes, were generated using the 10× Genomics Visium Spatial Gene Expression platform (3′ v1 chemistry). In brief, cryosectioned tumor tissues were fixed, H&E‐stained, and imaged prior to permeabilization and reverse transcription on spatially barcoded capture slides. Libraries were prepared according to the manufacturer's protocol and sequenced on an Illumina platform at sufficient depth to resolve complex TME. Raw FASTQ files were processed with Space Ranger (v1.1.0) to align reads to the human reference genome (GRCh38), generating gene‐by‐spot count matrices. All subsequent analyses were performed using Scanpy (v1.9.1), a Python‐based single‐cell and spatial transcriptomics toolkit.

### Preprocessing, Normalization, and Dimensionality Reduction

Initial quality control filtered out spatial barcodes (spots) with low gene detection (<200 genes) and high mitochondrial gene content (>20%), as well as genes expressed in fewer than three spatial locations. Counts were normalized per spot to a uniform depth and log‐transformed. Highly variable genes were selected for dimensionality reduction via PCA. The top 20 principal components were retained for graph‐based clustering using the Leiden algorithm at a resolution of 1.0. UMAP was used for visualizing transcriptomic domains.

### Spatial Enhancement and Feature Detection

To enhance spatial resolution and preserve local tissue structure, we applied spatial neighborhood‐based smoothing using the Squidpy framework, a companion toolkit for spatial omics within the Scanpy ecosystem. Tissue architecture was refined through spatial graph construction and domain‐level smoothing, enabling improved detection of local transcriptional gradients.

### Signature Scoring and Spatial Mapping

To annotate spatially localized functional phenotypes within the TME, we computed module scores for a curated HLA‐DR⁺ tumor cell gene signature, which reflects transcriptional programs associated with antigen presentation by malignant cells. Gene signature scoring was performed using the score_genes function from the Scanpy toolkit, which calculates the expression score for a set of target genes in each spatial spot by computing the average expression of the signature genes, followed by subtraction of the average expression of a randomly sampled reference gene set matched for expression distribution. This normalization strategy mitigates potential biases from technical variability and local gene expression noise. The resulting module scores were then projected back onto the tissue's spatial grid to visualize regional enrichment of HLA‐DR⁺ tumor‐like transcriptional states. Spatial mapping revealed discrete tumor zones with elevated antigen‐presenting capacity, often colocalized with immune infiltration, suggesting a potential interface between tumor cells and effector immune populations. These patterns supported the presence of functionally specialized tumor subpopulations with distinct immunological roles.

### Statistical Analysis

All statistical analyses were performed using R (v4.0.2) and Python (v3.7.4) in JupyterLab (v4.0.6). Continuous variables were compared using the Student's *t*‐test or Wilcoxon rank‐sum test. Categorical variables were analyzed using the chi‐squared test or Fisher's exact test. *p*‐values were adjusted for multiple testing using the Benjamini‐Hochberg method. Unless otherwise stated, *p* < 0.05 was considered statistically significant.

### Ethics Statement

All human tissue specimens and associated clinical data were collected in accordance with protocols approved by the Medical Ethics Committee of Henan Provincial People's Hospital (Approval (2022) Ethical Review No. 175). All procedures were conducted in compliance with institutional ethical standards and the principles of the Declaration of Helsinki (1964) and its subsequent revisions. Written informed consent was obtained from each participant or their legal guardian prior to enrollment in the study.

## Conflict of Interest

The authors declared no conflict of interest.

## Supporting information



Supporting Information

Supplemental Table 1

## Data Availability

The data that support the findings of this study are openly available in Spatial transcriptomics and scRNA seq of liver tissue in human HCC at https://www.ncbi.nlm.nih.gov/sra/PRJNA998229, reference number [1].
