# Supplementary material for: HLA‐DR⁺ Tumor Cells Show an Association with a Distinct Immune Microenvironment and CD8⁺ T‐Cell Exhaustion in HBV‐Associated Hepatocellular Carcinoma
Source: Adv Sci (Weinh). 2025 Jun 4;12(30):e02979. doi: 10.1002/advs.202502979 (PMC12376660; doi:10.1002/advs.202502979)
Supplement: Supplementary file 1 — Supporting Information [file ADVS-12-e02979-s002.docx]

Supplementary Materials

Figure S1. Demographic, Clinical, and Gene Expression Patterns in hepatitis B virus (HBV)-, hepatitis C virus (HCV)-, and non-B non-C (NBNC)-Associated Liver Cancers. **(A)** Heatmap comparing the average expression of key genes across major cell subsets (NK cells, γδ T cells, CD4^+^ T cells, CD8^+^ T cells, B cells, mast cells, neutrophils, monocytes, dendritic cells, macrophages, and tumor cells). Warmer hues denote higher expression levels, while dendrogram clustering on the right groups similarly expressed genes. **(B--E)** Uniform Manifold Approximation and Projection (UMAP) projections of single-cell data annotated by four clinical variables: gender (B), viral etiology (C, HBV/HCV/NBNC), cancer type (D, including adenosquamous carcinoma (ASC), cholangiocellular carcinoma (CHC), colorectal cancer (CRC), intrahepatic cholangiocarcinoma (ICC), and hepatocellular carcinoma (HCC)), and Barcelona Clinic Liver Cancer (BCLC) stage (E). Each panel highlights how these features influence overall cellular distribution. **(F--I)** Stacked bar plots illustrating group distributions for gender (F), cirrhosis status (G), BCLC stage (H), and cancer type (I) within each viral etiology cohort. The relative proportions underscore clinical differences among HBV-, HCV-, and NBNC-associated patient groups.


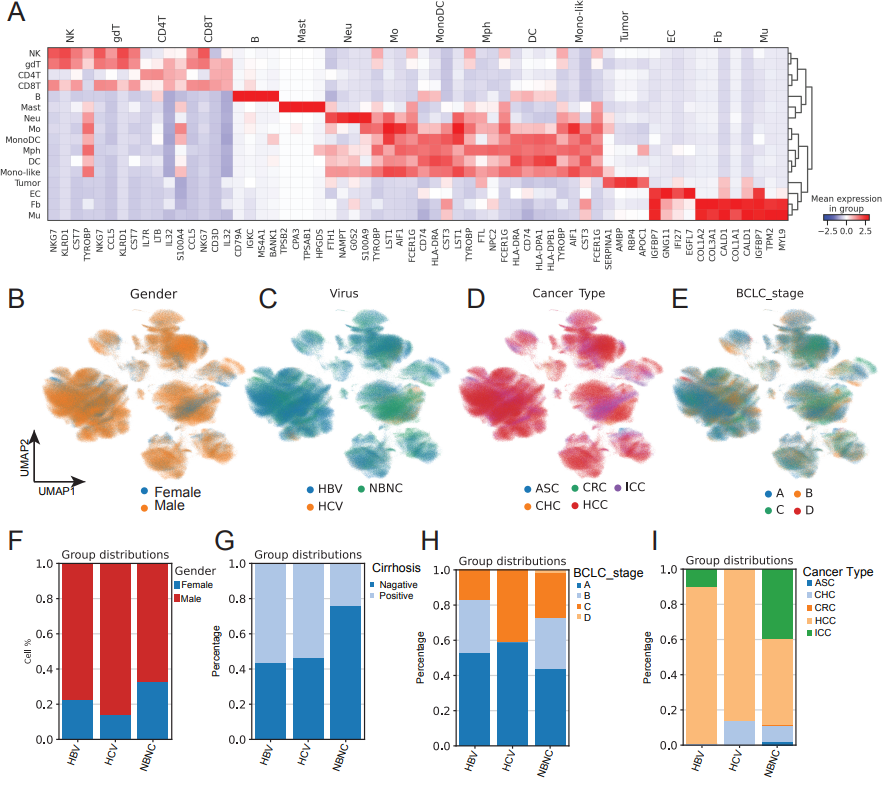


Figure S2. Comparative Analysis of Cell-Type Correlations, Histology, and Ligand--Receptor Interactions in hepatitis B virus (HBV)-, hepatitis C virus (HCV)-, and non-B non-C (NBNC)-Associated hepatocellular carcinoma (HCC). **(A)** Correlation matrix depicting interactions among cell subsets from HBV, HCV, and NBNC samples. The heatmap ranges from −1.0 to +1.0, with red and blue representing strong positive and negative correlations, respectively. Rows and columns are hierarchically clustered to highlight shared expression patterns across cell types. **(B)** Representative hematoxylin and eosin (H&E)--stained tissue microarray cores for HBV, HCV, and NBNC tumors. Each core shows characteristic histological features associated with its etiological background. **(C)** Representative multiplex immunofluorescence images of tissue microarray cores from HBV (Top), HCV (Middle), and NBNC (Bottom) tumors. Fluorescent channels include DAPI (nuclei, blue), PanCK (tumor cells, cyan), CD8 (green). **(D)** Chord diagrams illustrating key ligand--receptor interactions among major cell subsets (B cells, tumor cells, endothelial cells, CD4^+^ T cells, CD8^+^ T cells, macrophages, dendritic cells, mast cells, neutrophils, Natural cells (NK), fibroblasts, and others) in HCV (left) and NBNC (right) cohorts. The thickness of each chord corresponds to the interaction strength, with colored arcs indicating specific receptor--ligand pairs that shape tumor-immune crosstalk.


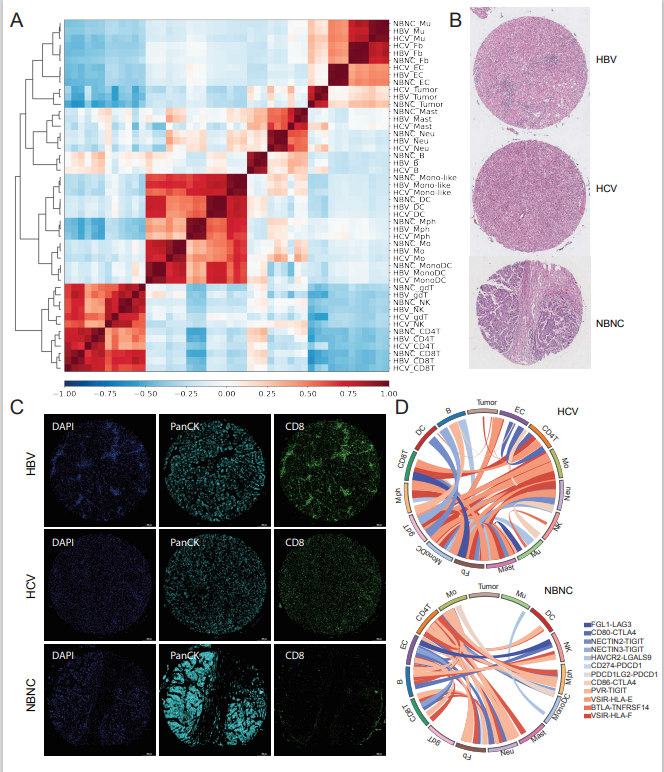


Figure S3. Pathway Signatures, Copy Number Variation, and HLA Gene Expression in hepatitis B virus (HBV)-, hepatitis C virus (HCV)-, and non-B non-C (NBNC)-Associated Tumor Cells. **(A)** Bar plots showing pathway or gene signatures for apoptosis, G2M, p53, Myc, and Kras in tumor cells from HBV, HCV, and NBNC cohorts. Values represent normalized scores, with statistical comparisons indicated. **(B)** Heatmap of inferred copy number variations (inferCNV) across chromosomes 1 to 22, highlighting amplifications (red) and deletions (blue) in HBV-, HCV-, and NBNC-derived tumor cells compared to reference controls. **(C)** Summarized CNV scores from inferCNV analysis for each etiological group (HBV, HCV, NBNC). Bars represent mean values, and asterisks denote significance levels. **(D)** Bubble plot illustrating HLA gene expression (*HLA-A, -B, -C, -F*, etc.) in HBV, HCV, and NBNC tumor cells. Bubble size corresponds to the fraction of cells expressing each gene, while color intensity indicates mean expression within each group.


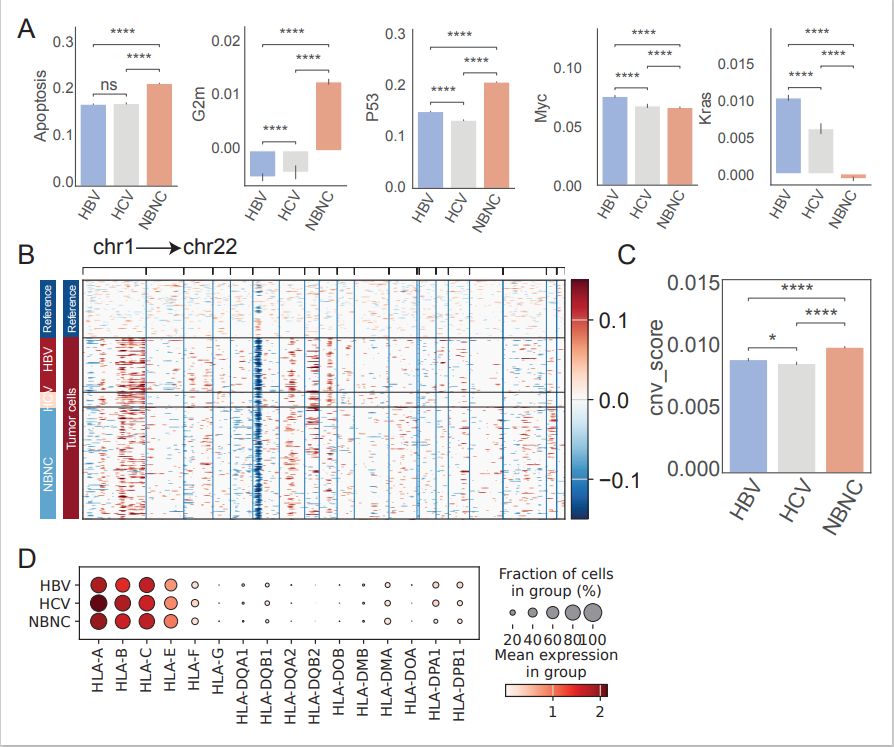


Figure S4. HLA-DR and PD-L1 Expression in hepatitis B virus (HBV), hepatitis C virus (HCV), and non-B non-C (NBNC) Tumor Cells. **(A)** Representative flow cytometry dot plots showing HLA-DR versus PanCK (top row) and PD-L1 versus PanCK (bottom row) in HBV, HCV, and NBNC tumors. Percentages denote the fraction of cells in each quadrant, highlighting HLA-DR^+^ or PD-L1^+^ populations within PanCK^+^ tumor cells. **(B)** Representative multiplex immunofluorescence (mIF) images of tissue microarray（TMA) cores from HBV (Top), HCV (middle), and NBNC (Botto) tumors. Fluorescent channels include DAPI (nuclei, blue), PanCK (tumor cells, cyan), CD8 (green), and HLA-DR (yellow). **(C)** Gene set enrichment analysis comparing PD-L1 pathway expression in tumor cells with high versus low HLA-DR levels (NES = 1.46, *P* = 0.02). **(D)** Additional gating plots for PD-L1 expression among HLA-DR^+^PanCK^+^ tumor cells across HBV, HCV, and NBNC groups, illustrating etiological differences in co-expression of antigen-presentation and immune-checkpoint markers. **(E)** Representative mIF images of TMA sections from HBV (Top), HCV (Middle), and NBNC (Bottom) tumors. Panels show single fluorescence channels: DAPI (nuclei, blue), PanCK (tumor cells, cyan), PD-L1 (magenta), and HLA-DR (yellow).


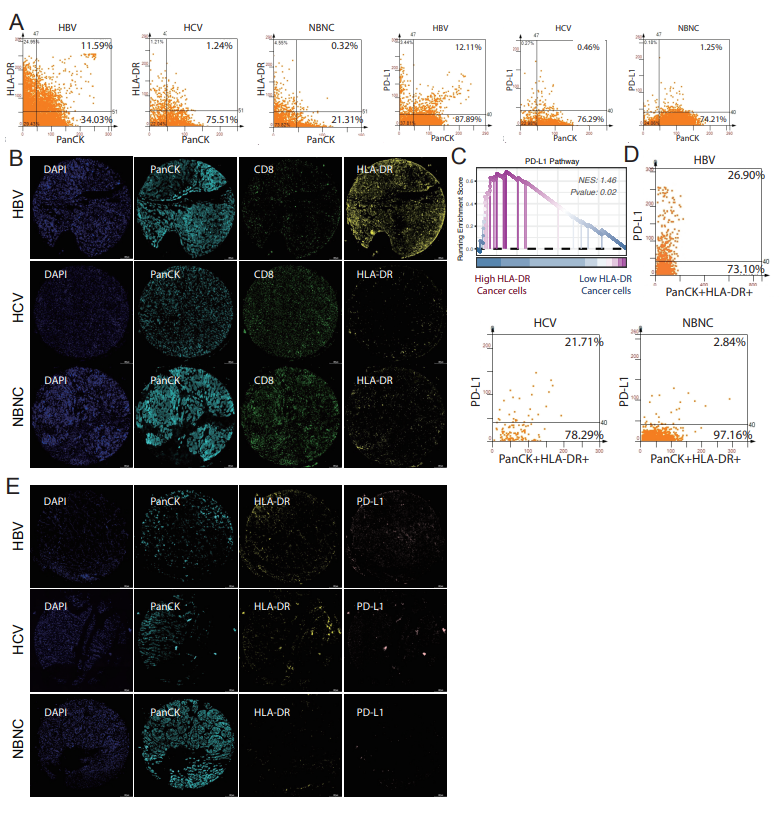


Figure S5. Further Characterization of CD8^+^ T Cell Subsets. **(A)** Scatter plot showing CD8A versus CD3D expression used to identify the CD8^+^ T cell population from single-cell data. **(B)** Bar chart quantifying the number of cells assigned to each CD8^+^ T cell subset (labeled 1--7), including mitotic cells, exhausted cells (TEX), effector memory cells (TEM), MAIT cells, central memory cells (TCM), terminal effector cells (TEMRA/TEFF), and naïve cells (TN). **(C)** Heatmap of pairwise transcriptomic similarity among these subsets, with hierarchical clustering revealing closely related cell phenotypes. **(D)** Uniform Manifold Approximation and Projection (UMAP) overlays illustrating the expression of representative marker genes (e.g., *CCR7, LEF1, SELL, TCF7, PRF1, GZMK, IFNG,* and others) across the CD8^+^ T cell landscape. Warmer colors indicate higher expression, highlighting distinct functional attributes within each subset.


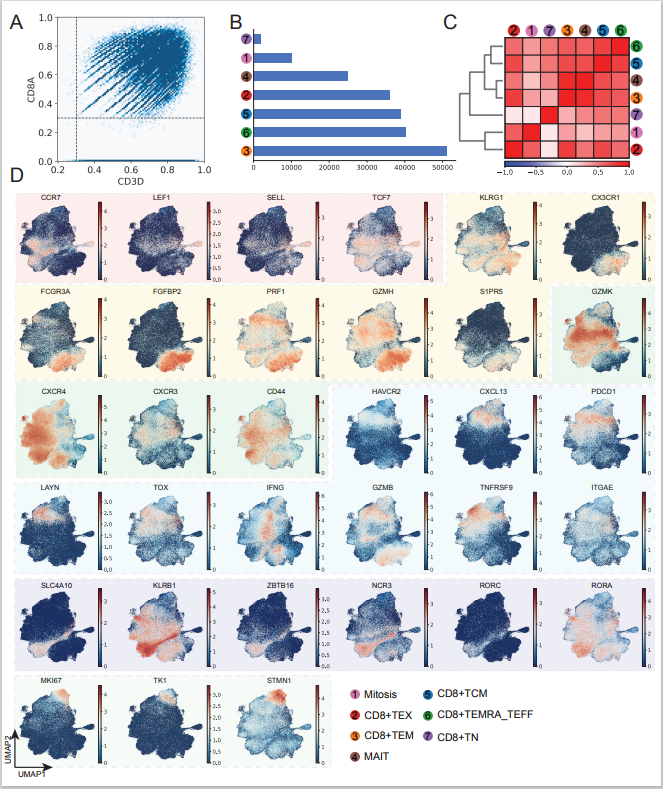


Figure S6. RNA Velocity--Based Pseudotime Analysis of CD8^+^ T Cells Across hepatitis B virus (HBV), hepatitis C virus (HCV), and non-B non-C (NBNC). **(A)** Uniform Manifold Approximation and Projection (UMAP) projection of CD8^+^ T cells annotated by viral etiology (blue for HBV, orange for HCV, green for NBNC), illustrating overall distribution of each group. **(B)** Stacked bar plots showing the proportional composition of CD8^+^ T cell subsets ( exhausted cells (TEX), central memory cells (TCM), effector memory cells (TEM), terminal effector cells (TEMRA/TEFF), naïve cells (TN), MAIT cells, and mitotic cells) in HBV-, HCV-, and NBNC-infected samples. **(C)** Representative multiplex immunofluorescence (mIF) images of TMA sections from HBV (Top), HCV (Middle), and NBNC (Bottom) tumors. Panels show single fluorescence channels: DAPI (nuclei, blue), PanCK (tumor cells, cyan), CD8 (green), CTLA4 (orange), and GZMB (granzyme B, red). **(D)** UMAP colored by RNA velocity--derived pseudotime, ranging from early (purple) to late (yellow) in the trajectory, indicating the inferred differentiation path of CD8^+^ T cells. **(E)** Density plots showing the distribution of CD8⁺ T-cell subsets along RNA velocity--derived pseudotime trajectories in HBV (Top), HCV (Middle), and NBNC (Bottom) HCC patients. Each colored region represents a distinct CD8⁺ T-cell subset, including CD8⁺ TCM (central memory), CD8⁺ TEM (effector memory), CD8⁺ TEMRA_TEFF (effector-like TEMRA), CD8⁺ TEX (exhausted), CD8⁺ TN (naïve), MAIT (mucosal-associated invariant T cells), and proliferating (mitotic) cells. The y-axis indicates cell density at each point along the pseudotime trajectory, reflecting the relative abundance of each subset. Differences in subset composition and transitions across pseudotime highlight infection-specific trajectories of CD8⁺ T-cell differentiation and exhaustion dynamics.


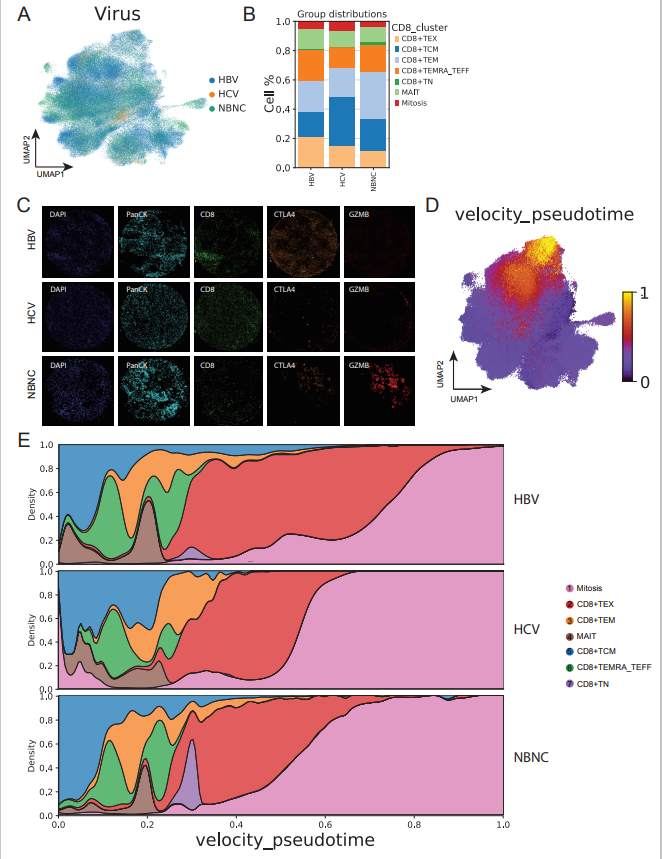


**Figure S7. Additional Analyses of HLA-DR^+^ Tumor Cells and CD8^+^ T-Cell Subtypes** **(A)** Radar plots for each CD8^+^ T-cell subset (naïve cells (TN), central memory cells (TCM), effector memory cells (TEM), exhausted cells (TEX), terminal effector cells (TEMRA/TEFF), mitotic cells and MAIT cells) comparing expression of immunoregulatory and cytotoxic genes in high versus low HLA-DR tumors. **(B)** Bar charts showing that T cell receptor signaling upregulation is most pronounced in hepatitis B virus (HBV)-associated HCC among high HLA-DR tumors, with parallels in hepatitis C virus (HCV) and non-B non-C (NBNC) groups.


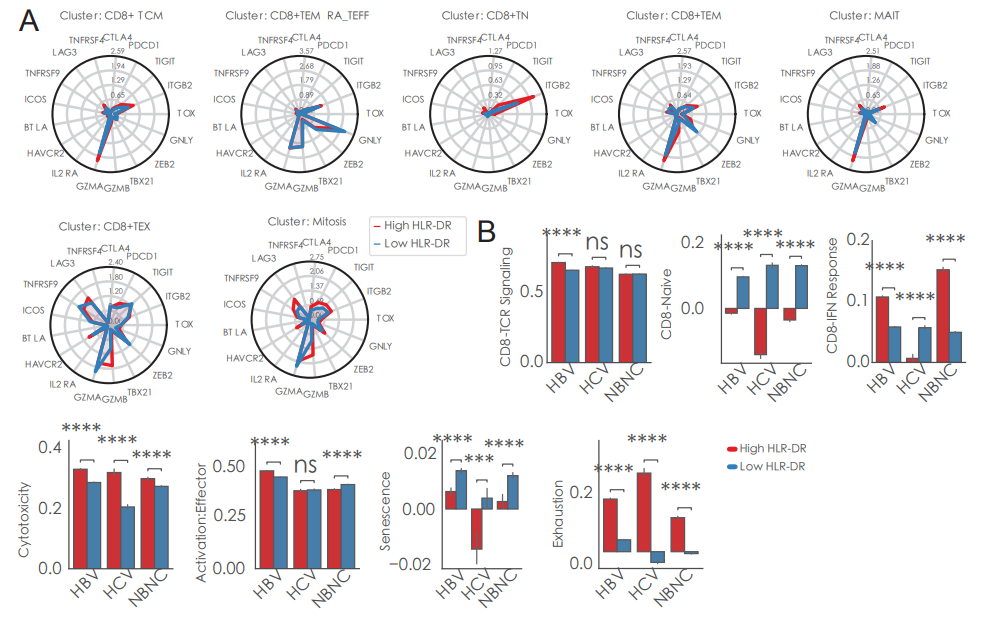


Figure S8. HLA-DR+ Tumor Cells and Response to Immunotherapy. **(A)** Kaplan--Meier survival curves comparing hepatocellular carcinoma (HCC) patients stratified by hepatitis B virus (HBV), hepatitis C virus (HCV), or non-B non-C (NBNC) viral status. The left and middle panels depict overall survival (OS) and progression-free survival (PFS), respectively, from the LIHC Virus Dataset (n = 729). The right panel illustrates OS from the TCGA-LIHC cohort. Despite the indicated hazard ratios (HR) and 95% confidence intervals (CI), no statistically significant survival differences were observed among the three etiological groups (all *P* > 0.05). **(B)** Representative contrast-enhanced CT scans of HBV, HCV, and NBNC patients before and after immunotherapy (A) or immunotherapy combined with targeted therapy (B). HBV^+^HCC cases show more pronounced tumor shrinkage and enhanced response compared to HCV and NBNC cohorts, aligning with elevated HLA-DR^+^ tumor-cell signatures.


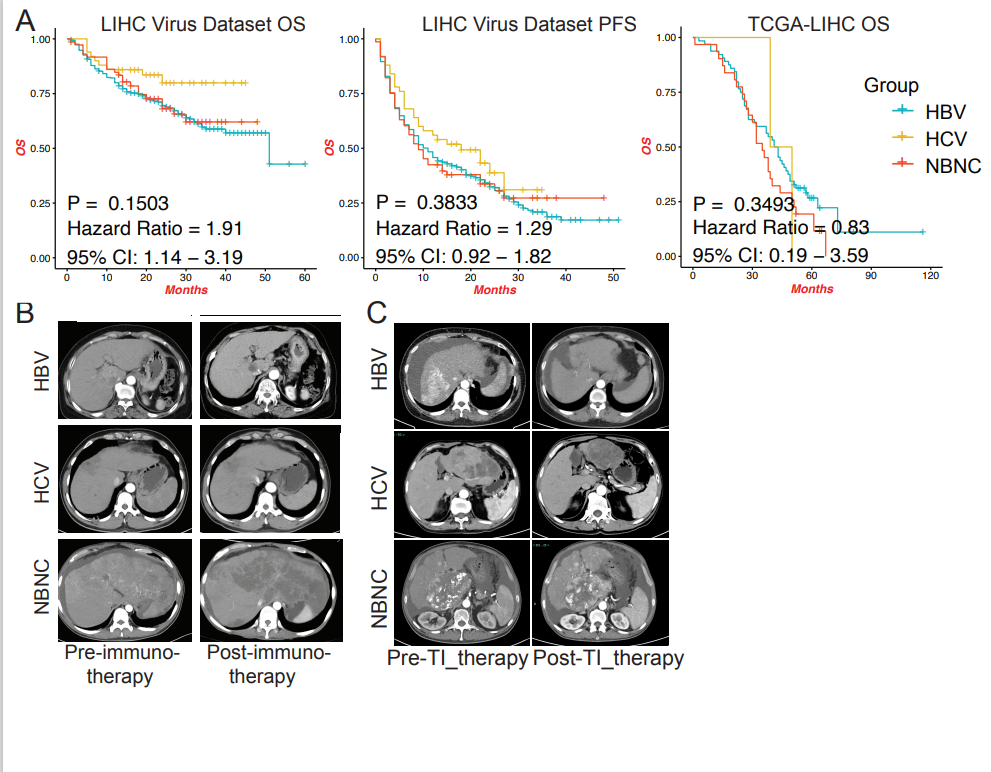


**Table S1:** Clinical and pathological data from 729 hepatocellular carcinoma (HCC) patients treated at Henan Provincial Hospital between 2020 and 2025.
